# Machine learning-based identification of factors associated with spontaneous abortion in patients with Systemic lupus erythematosus (SLE): Insights from the Egyptian College of Rheumatology (ECR)–SLE cohort

**DOI:** 10.1177/09612033261415984

**Published:** 2026-01-11

**Authors:** Nevin Hammam, Walaa N Ismail, Iman I El-Gazzar, Noha M Khalil, Eman F Mohamed, Nermeen Noshy, Dina F El-Essawi, Osman Hammam, Rawhya R El-Shereef, Faten Ismail, Marwa ElKhalifa, Hanan M Fathi, Soha Senara, Samah Ismail Nasef, Amany R El-Najjar, Ahmed M Abdalla, Ali Bakhiet, Ahmed M ElSaman, Mohamed Ismail Abdelkareem, Samar Tharwat, Tamer A Gheita

**Affiliations:** 1 Rheumatology Department, Faculty of Medicine, 68797 Assuit University, Assuit, Egypt; 2 Faculty of Computers and Information, 68843 Minia University, Minia, Egypt; 3 Department of Management Information Systems, College of Business, Al Yamamah University, Riyadh, Saudi Arabia; 4 Rheumatology Department, Faculty of Medicine, 63527 Cairo University, Cairo, Egypt; 5 Internal Medicine Department, Rheumatology Unit, Faculty of Medicine, Cairo University, Cairo, Egypt; 6 Internal Medicine Department, Rheumatology Unit, Faculty of Medicine (Girls), 590966 Al-Azhar University, Cairo, Egypt; 7 Internal Medicine Department, Rheumatology Unit, Faculty of Medicine, 68792 Ain-Shams University, Cairo, Egypt; 8 Internal Medicine Department, Rheumatology Unit, Egyptian Atomic Energy Authority (EAEA), Cairo, Egypt; 9 Department of Rheumatology and Rehabilitation, Faculty of Medicine, 612860 New Valley University, New Valley, Egypt; 10 Rheumatology Department, Faculty of Medicine, 68843 Minia University, Minia, Egypt; 11 Internal Medicine Department, Rheumatology Unit, Faculty of Medicine, 68789 Alexandria University, Alexandria, Egypt; 12 Rheumatology Department, Faculty of Medicine, 158405 Fayoum University, Fayoum, Egypt; 13 Rheumatology Department, Faculty of Medicine, Suez-Canal University, Ismailia, Egypt; 14 Rheumatology Department, Faculty of Medicine, 68865 Zagazig University, Sharkia, Egypt; 15 Rheumatology Department, Faculty of Medicine, 435387 Aswan University, Aswan, Egypt; 16 Higher institute of Computer Science and Information Systems, 501281 Culture & Science City, Giza, Egypt; 17 Rheumatology Department, Faculty of Medicine, 68889 Sohag University, Sohag, Egypt; 18 Rheumatology and Rehabilitation Department, 486470 Al-Azhar Faculty of Medicine, Assiut, Egypt; 19 Rheumatology Unit, Internal Medicine, 68780 Mansoura University, Dakahlia, Egypt

**Keywords:** Spontaneous abortion, systemic lupus erythematosus, machine learning

## Abstract

**Background:**

Systemic lupus erythematosus (SLE), an autoimmune disease, predominantly affects women and is associated with an increased risk of spontaneous abortion (SA). However, traditional analytical methods found a modest relationship between some factors and SLE-SA and were limited to a small sample size, frequently associated with poor predictive performance.

**Objectives:**

This study aimed to apply and evaluate an Extreme Gradient Boosting (XGBoost) model using routinely collected clinical data to identify patterns associated with spontaneous abortion in women with SLE and to identify the key variables associated with this outcome.

**Methods:**

The study included adult SLE women from the Egyptian College of Rheumatology (ECR)-SLE cohort, a national multicenter study, which had available SA data. SA was defined as unexplained pregnancy loss up to 20 weeks of gestation. Patients’ demographics, clinical manifestations, SLE disease activity index (SLEDAI), therapeutic and laboratory data were used as input variables for the logistic regression (LR) and XGBoost models. We evaluated the performance of both the XGBoost and LR models by calculating the area under the receiver operating characteristic curve (AUC) for each model, and then compared these AUC values to assess which model better distinguished between patients with and without SA. The importance and direction of each variable contributing to the risk of SA were evaluated using SHapley Additive exPlanation (SHAP).

**Results:**

A total of 3296 SLE women (mean ± SD age: 32.5 ± 10.1 years; median disease duration: 48 months) were included. The mean SLEDAI score was 11.3 ± 9.5. About 13.9% of the patients included had at least one abortion. Optimized XGBoost performed better (AUC 0.99) compared with LR (AUC 0.78). Positive antiphospholipid antibodies, low complement 3, longer disease duration, hypertension and the presence of mucocutaneous ulcers, as well as anticoagulants and steroid use, were among the important factors associated with SA in SLE patients.

**Conclusion:**

Using information obtained in the clinical settings, the XGBoost identified variables associated with SA in women with SLE, including positive antiphospholipid antibodies, low complement 3 levels and longer disease duration. Further, longitudinal studies are necessary to evaluate the clinical utility of the proposed classification model.

## Introduction

Systemic lupus erythematosus (SLE) is a chronic autoimmune inflammatory disease with multi-organ involvement that preferentially affects women, with a female-to-male ratio of 10:1.^1^ This significant disparity suggests that hormonal and genetic factors may play a crucial role in the disease’s prevalence among women. As SLE affects predominantly women of childbearing age, pregnancy and family planning are topics of key interest both in research and in clinical practice.^2^

Spontaneous abortion (SA) is characterized by the unintentional fetal loss before 20 weeks of gestation.^3^ The risk of pregnancy loss is about 20% in women with SLE. Having an abortion is a traumatic experience that can have adverse effects on the mother.^4^ The complex pathogenesis of SA is impacted by a multitude of biological, socioeconomic, and lifestyle factors, which exhibit substantial variation across populations.^5^ Several studies had shown that some clinical and laboratory indicators could predict the risk of SA in SLE women.^6–8^ Pregnancy complications among SLE patients were found to be associated with renal involvement, anti-dsDNA, antiphospholipid antibody, anti-Ro/SSA, increased ESR, and a younger age at disease onset.^6^

Identifying risk factors for SA is necessary to improve maternal and fetal outcomes. Considering the traditional statistical method to predict pregnancy adverse outcomes, many studies have been conducted worldwide to identify these factors. For example, factors such as lupus nephritis, high blood pressure, thrombosis, anti-phospholipid antibodies, anemia, leukopenia, history of abortion, and many other factors have been reported.^6–9^ However, the utilized models found a modest correlation between clinical and laboratory factors, including anti-dsDNA positivity, antiphospholipid antibody positivity, anti-Ro/SSA, and elevated ESR and SLE-SA, were limited to small sample sizes relative to the number of variables and were frequently associated with poor predictive performance upon validation. The numerous risk factors and the complexity of relationships among these risk factors advocate for applying a machine learning (ML) approach to handle this complexity better than traditional statistical models.

Machine learning (ML) techniques are characterized by their ability to analyze vast amounts of data rapidly and accurately and uncover relationships between hidden patterns and symptoms, allowing robust analysis.^10^ Recent studies have explored the potential of ML to predict pregnancy outcomes in SLE cohorts, particularly in settings with limited sample sizes. For example, prior work demonstrated that ML models can effectively analyze small datasets (e.g., 51 pregnancies with 288 variables) to identify risk factors and compensate for statistical limitations inherent to traditional methods.^11^ The Extreme Gradient Boosting (XGBoost) is one of the ML algorithms that can effectively captures nonlinear relationships and interactions between features and can identify key predictive factors associated with different medical conditions.

Although previous studies have established that women with SLE face an increased risk of spontaneous abortion, the precise risk remains variable across populations and is influenced by both general and SLE-specific factors.^12,13^ Established risk factors for abortion in SLE include renal involvement, antiphospholipid antibody positivity, low complement 3, longer disease duration, hypertension, and the presence of mucocutaneous ulcers, as well as the use of steroids and anticoagulants.^14,15^ Despite advances in clinical care, traditional statistical models have been limited in their ability to predict individual risk due to the complex interplay of these variables and often modest predictive performance.^16^ To overcome these limitations, recent research has turned to ML methods, which can handle large datasets and complex, nonlinear relationships among predictors.^16^

Using data already collected during standard healthcare clinical practice in research is important because it allows researchers to access large, real-world dataset that reflect typical patient populations, providing insights into disease patterns while minimizing the burden on patients and reducing research costs. To the best of our knowledge, application of ML using a large SLE dataset in identifying the risk factors for SA using routine clinical data has not been previously reported. Thus, the aim of this study was to apply and evaluate an Extreme Gradient Boosting model using routinely collected clinical data to identify patterns associated with spontaneous abortion in women with SLE and to identify the key variables associated with this outcome.

## Methods

### Study design and study population

This is a cross-sectional study. Data were obtained from specialized rheumatology departments and centers representing governorates across Egypt by the members of the Egyptian Colleague of Rheumatology-Systemic Lupus Erythematosus (ECR-SLE) study group.^17^ The data were locked on January 2023. Records were then filtered based on the following criteria: (a) female patients (b) diagnosis of SLE according to systemic lupus international collaborating clinics (SLICC) classification criteria^18^; (c) age >18; and (d) available data about SA. Exclusion criteria included patients with SLE who had pregnancies with medically induced abortion or a miscarriage that was caused by an aberrant chromosome karyotype of the fetus.

### Ethical consideration

The study was carried out following the principles outlined in the Helsinki Declaration,^19^ and the Institutional Research Board of the Faculty of Medicine at Mansoura University provided its approval (R.24.05.2630) to the study protocol.

### Baseline demographic and clinical data

The following data were obtained from the patients: age and disease duration. The measurements of weight and height were taken, and subsequently, the body mass index (BMI) was computed. A comprehensive history and clinical evaluation were conducted, paying particular attention to the presence of current mucosal ulcers, cutaneous, ocular, neurological, gastrointestinal, and musculoskeletal manifestations, as well as vasculitis, cardiovascular, pulmonary, and renal disease defined as in Petri et al.^18^ Patients were assessed for lupus activity by SLE disease activity index (SLEDAI) score.^20^ The assessment of irreversible organ damage was conducted using the Systemic Lupus International Collaborating Clinics (SLICCs)/American College of Rheumatology (ACR) Damage Index (SDI).^21^

### Therapeutic data

Data pertaining to current therapeutic interventions was collected from the electronic or printed medical records of every participant. The researchers gathered data on the current medications administered by participants, including antimalarials, corticosteroids, cyclophosphamide, azathioprine, cyclosporine A, methotrexate, mycophenolate, biologics, and anticoagulants or low dose aspirin (LDA).

### Laboratory assessment

On the days of clinical examination, a blood sample was drawn from each patient, and relevant laboratory tests were assessed. The laboratory tests included a complete blood count (CBC), serum creatinine, erythrocyte sedimentation rate (ESR), C-reactive protein (CRP), 24 hr urinary protein, antiphospholipid antibodies (aPL), anti-nuclear antibody (ANA), anti-double standard DNA (anti ds-DNA) antibody, as well as complement 3 and 4. The presence of antiphospholipid syndrome (APS) has been determined based on the Sydney criteria for antiphospholipid syndrome.^22^

### Study groups (target outcome)

Patients were classified into two groups: those with no previous history of SA, and those with a history of at least one SA. The term “SA” refers to a medically acknowledged occurrence of embryonic or fetal death or the passing of products of conception before the 20th week of gestation, without any external intervention.^23^

### Statistical analysis

Statistical analysis was conducted using Stata statistical software version 15 (Stata-Corp) and Python language (version 3.7.12). Normally distributed variables were summarized using the mean ± standard deviation (SD), and non-normally distributed variables by the median and interquartile range (IQR). Frequencies were expressed by percentage. Mean characteristics between patients with and without SA were compared using a two-sample t-test, and proportions were compared using the chi-square test. Two-sided *p* < 0.05 was considered statistically significant.

### Extreme gradient boosting (XGBoost) approach

XGBoost approach involved the following steps: data pre-processing, variables (features) selection, data splitting, model development, model evaluation, feature importance derivation, and interpretation. Data pre-processing and features selection: Data had an overall good quality. Demographic, clinical, laboratory, and therapeutic data of relevance were selected based on clinical expertise and literature review and considered as the features for the process. Applying feature selection technique before training XGBoost models is a recognized practice in ML, aimed at enhancing model performance and interpretability. Features were evaluated based on their frequency of use and their impact on reducing impurity or entropy. A final step involves iteratively selecting features and validating the selection process to ensure the selected features are generalizable to future data and to prevent overfitting. As a result, 47 candidate variables were selected as the input features for XGBoost. For the included variables with some data missing, regressive imputation was trained to predict the observed values of a variable based on other variables in the dataset, and the model was then used to impute the missing values of the variable. Prior to the model development, the dataset was randomly divided into training (80%) and testing (20%) data. Afterward, the XGBoost model was trained using the training set, then evaluated on the testing data.

Data splitting was performed prior to imputation to prevent data leakage. Imputation models were fitted on the training set and applied to the test set. For categorical variables, k-nearest neighbor imputation was applied. The number of neighbors for kNN imputation was set to k = 5 (default setting), and sensitivity checks with alternative k values confirmed comparable results^24^; for continuous variables, we used a linear regression model. Cross-validation was performed during the training phase to ensure robust model evaluation. Specifically, k-fold cross-validation was used to assess the generalizability of the model. In the current work, a 10-fold cross-validation was conducted, the data was split into 10 subsets, with the model being trained on four subsets and validated on the remaining one in each iteration. This process was repeated for each fold, and the results were averaged to provide a more reliable estimate of the model’s performance. In addition, hyperparameter tuning involved training multiple models across different hyperparameter combinations to find the best model. The testing set was used to evaluate the final performance of the model. To achieve superior predictive performance and higher classification accuracy, the Harris Hawks (HH) Optimization algorithm explored multiple hyperparameter combinations, retraining the model iteratively to determine the optimal parameter in the XGBoost model (HH-XGB).^25^

For model evaluation, the receiver operating characteristic curve (AUC) was used. Additionally, the performance of the model was evaluated by accuracy, sensitivity, specificity, negative predictive value (NPV), and positive predictive value (PPV) and compared to the conventional LR method. LR, representing the simplest of all conventional classifiers, was chosen to create a reference model against the performance of HH-XGB.

As the final step, SHapley Additive exPlanation (SHAP) values were used to interpret the XGBoost model and identify the contribution of each feature to the predictions.^26^ SHAP is a method for explaining the output of a machine learning model, to approximate the importance and direction of each feature in a model.

## Results

### Characteristics of the patients’ cohort

In total, there were 3296 SLE women who participated in the study, all of whom were from multiple tertiary rheumatology centers all over Egypt. Their mean age was 32.5 ± 10.1 years with a median disease duration of 48 months. According to the presence of a previous history of at least one SA, a total of 2837 patients were assigned to the non-SA group, whereas 459 (13.92%) were assigned to the SA group. Table 1 illustrates the clinical and therapeutic features of the two groups.

**Table 1.** Clinal features and therapeutic data of the study SLE patients (*n* = 3296).

Variable Mean ± SD or median (IQR) or *N* (%)	Total SLE patients (*n* = 3296)	SLE without SA (*n* = 2837)	SLE with SA (*n* = 459)	*p* value
Demographic data				
Age, years	32.55 (10.06)	32.37 (10.28)	33.58 (8.52)	**0.018**
Disease duration, months	4 (2, 7)	4 (2, 7)	5 (3, 8)	0.051
Body mass index, kg/m^2^	27.11 ± 5.45	26.88 ± 5.47	28.28 ± 5.20	**0.002**
Clinical features				
Mucous ulcer	231 (7.01)	148 (5.22)	83 (18.08)	**<0.0001**
Cutaneous manifestations	2253 (68.36)	1928 (67.96)	325 (70.81)	0.224
Ocular manifestations	299 (9.07)	256 (9.02)	43 (9.37)	0.812
Musculoskeletal manifestations	2427 (73.63)	2088 (73.60)	339 (73.86)	0.908
Neurological manifestations	732 (22.21)	635 (22.38)	97 (21.13)	0.550
Vasculitis	282 (8.56)	231 (8.14)	51 (11.11)	**0.035**
Gastrointestinal manifestations	454 (13.77)	394 (13.89)	60 (13.07)	0.638
Cardiovascular disease	531 (16.11)	460 (16.21)	71 (15.47)	0.687
Pulmonary disease	858 (26.03)	734 (25.87)	124 (27.02)	0.605
Renal disease	1572 (47.69)	1343 (47.34)	229 (49.89)	0.310
Antiphospholipid syndrome	257 (17.33)	155 (12.59)	102 (40.48)	**<0.0001**
SLEDAI	11.34 ± 9.52	11.31 ± 9.38	11.51 ± 10.26	0.715
SLICCDAI	2.34 ± 2.54	2.27 ± 2.36	2.71 ± 3.34	0.094
Diabetes mellitus	221 (7.18)	164 (6.19)	57 (13.29)	**<0.0001**
Hypertension	569 (18.01)	404 (14.91)	165 (36.67)	**<0.0001**
Therapeutic data				
Hydroxychloroquine	2189 (66.41)	1812 (63.87)	377 (82.14)	**<0.0001**
Glucocorticoids	1940 (58.86)	1555 (54.81)	385 (83.88)	**<0.0001**
Cyclophosphamide	811 (24.61)	655 (23.09)	156 (33.99)	**<0.0001**
Azathioprine	1415 (42.93)	1173 (41.35)	242 (52.72)	**<0.0001**
Cyclosporine A	194 (19.4)	105 (21.4)	89 (17.5)	0.115
Methotrexate	120 (3.64)	99 (3.49)	21 (4.58)	0.249
Mycophenolate	350 (10.62)	290 (10.22)	60 (13.07)	0.066
Biologics	36 (1.09)	29 (1.02)	7 (1.53)	0.336
Anticoagulants	252 (7.65)	158 (5.57)	94 (20.48)	**<0.0001**
Low dose aspirin	180 (5.46)	115 (4.05)	65 (14.16)	**<0.0001**
Laboratory data				
Haemoglobin (g/dl)	10.43 ± 2.05	10.40 ± 2.09	10.56 ± 1.83	0.149
TLC (x10^3^/mm^3^)	6.1 (4.4, 8.6)	6 (4.4, 8.6)	6.5 (4.4, 8.5)	0.075
Platelets count (x10^3^/mm^3^)	239.02 ± 100.52	238.94 ± 101.63	239.45 ± 94.70	0.925
ESR (mm/1^st^hr)	50 (30, 80)	50 (30, 80)	54 (30, 87)	0.304
CRP titre (mg/L)	0.50 (0.50)	0.49 (0.50)	0.55 (0.50)	0.102
Serum creatinine (mg/dL)	0.8 (0.6, 1.1)	0.8 (0.6, 1.1)	0.8 (0.6, 1.2)	0.703
24 h proteinuria	0.45 (0.17, 1.25)	0.42 (0.16, 1.2)	0.58 (0.22, 1.4)	0.387
Positive APL	418 (22.75)	266 (17.62)	152 (46.48)	**<0.0001**
Positive ANA	3081 (93.48)	2642 (93.13)	439 (95.64)	**0.043**
Positive anti ds-DNA	1959 (77.13)	1634 (76.89)	325 (87.31)	0.529
Low complement 3	1117 (52.52)	932 (50.41)	185 (66.55)	**<0.0001**
Low complement 4	1083 (51.55)	932 (50.96)	151 (55.51)	0.160

SLEDAI: SLE disease activity index; SLICCDI: Systemic Lupus International Collaborating Clinics Damage Index; TLC: total leukocyte count; ESR: erythrocyte sedimentation rate; CRP: C-reactive protein; APL: antiphospholipid antibodies; ANA: anti-nuclear antibodies; and Anti-ds-DNA: anti double standard antibodies. Bold values are significant at p<0.05.

There was a statistically significant difference between the two groups regarding age (*p =* 0.018), mucosal ulceration (*p* < 0.0001), diabetes mellitus (*p* < 0.0001) and hypertension (*p* < 0.0001). Presence of vasculitis and positive antiphospholipid antibodies were significantly different between women with and without SA (*p* = 0.035, and *p* < 0.0001, respectively). Comparing therapeutic data of the two groups, we found that SA group had a higher rate of administration of hydroxychloroquine (82.14% vs 63.87%, *p* < 0.0001), glucocorticoids (83.88% vs 54.81%, *p* < 0.0001), cyclophosphamide (33.99% vs 23.09%, *p* < 0.0001), azathioprine (52.72% vs 41.35%, *p* < 0.0001), anticoagulants (20.48% vs 5.57%, *p* < 0.0001) and LDA (14.16 %vs 4.05%, *p* < 0.0001). SA group had a higher prevalence of positive APL (46.485% vs 17.62%, *p* < 0.0001) and low complement 3 (66.55% vs 50.41%, *p* < 0.0001).

### Performance of various ML models for classifying SA

**Figure 1.**
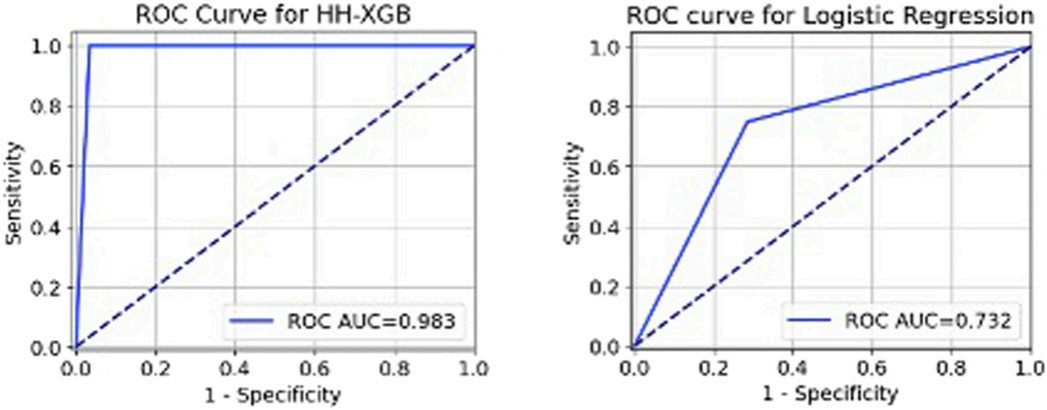
The ROC graph of the Harris Hawks (HH) Optimization XGBoost (HH-XGBoost) and Logistic regression for identifying spontaneous abortion (SA) in women with systemic lupus erythematosus. *Note. *Spontaneous abortion refers to a medically acknowledged occurrence of embryonic or fetal death or the passing of products of conception before the 20th week of gestation, without any external intervention.

The models’ discrimination performance is summarized in Table 2 and Figure 1. The AUC value for the conventional LR model was 0.784. In contrast, the HH-XGB model had superior performance in identifying SA patterns, achieving an overall AUC of 99% (AUC = 0.997). The HH-XGB model achieved higher sensitivity and specificity than LR and XGBoost (Table 2).

**Table 2.** Results of the performance of different models.

Models	Accuracy	F1-score	Sensitivity	Specificity	PPV	NPV	AUC
Logistic	0.731	0.732	0.752	0.711	0.715	0.749	0.732
XGBoost	0.945	0.945	1.0	0.899	0.894	1.0	0.947
HH-XGB	0.982	0.983	0.966	1.0	1.0	0.965	0.997

AUC: area under the curve, NPV: negative predictive value, PPV: positive predictive value, XGBoost: extreme gradient boosting, and Harris Hawks optimization XGBoost (HH-XGBoost).

### Feature importance and model interpretation

**Figure 2.**
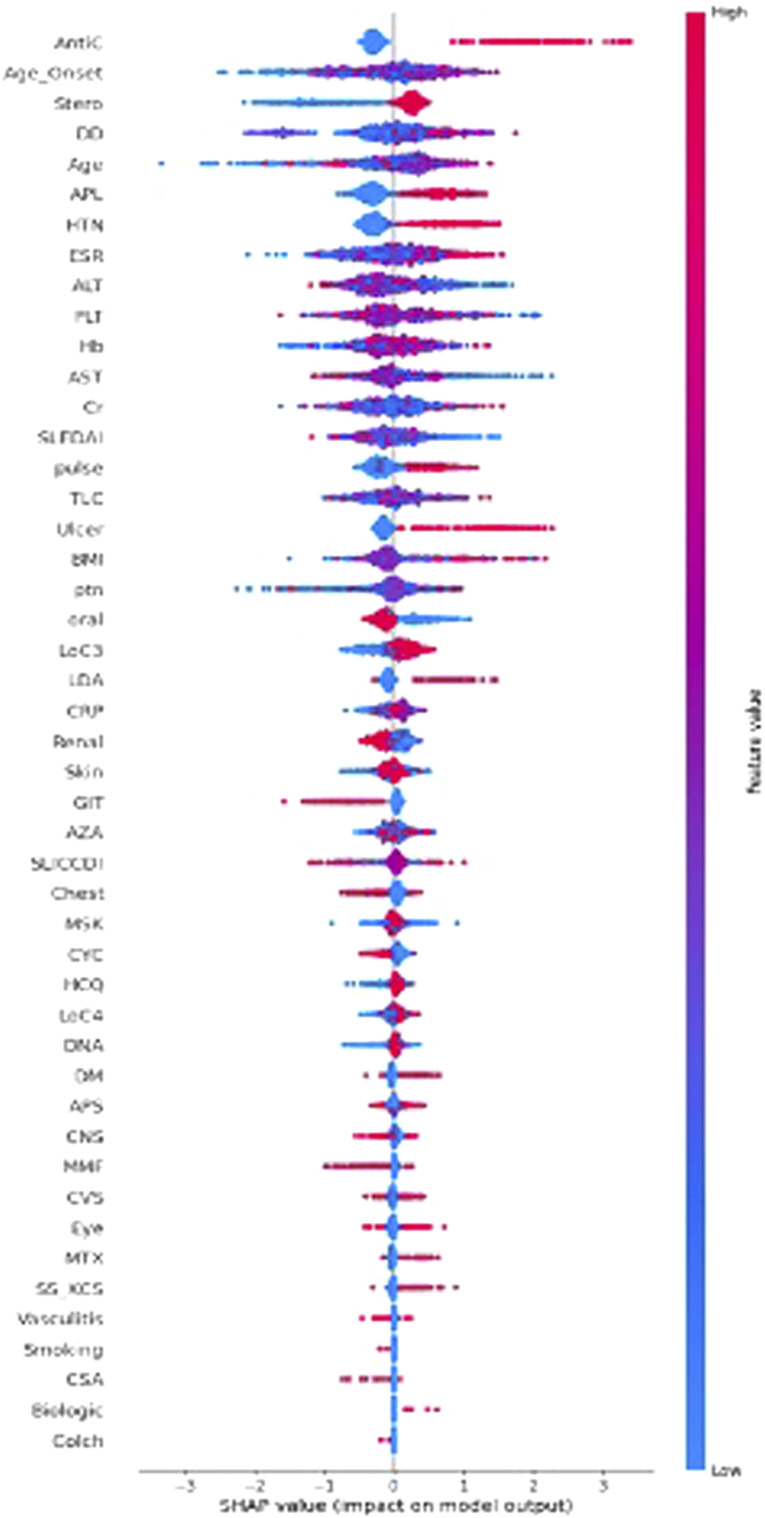
Overall SHapley Additive exPlanation (SHAP) values for the variables in Shapely plots to display both the feature importance and feature contribution to the model prediction. *Note. *Overall SHAP values for the variables in Shapely plots to display both the feature importance and feature contribution to the model prediction. Shapley plots show the SHAP values in the order of the important variables that contribute to spontaneous abortion. The *x*-axis represents the marginal contribution of a feature to the change in the predicted probability of development of spontaneous abortion. Colors indicate the value of the variable: red represents higher numerical values of the variable and blue represent lower numerical values. As all categorical variables were converted into binary indicators, zero (i.e., absence) is indicated with blue dots and one (i.e., presence) is represented by red dots.

Figure 2 shows the influence of variables on SA in the HH-XGB model. Positive antiphospholipid antibodies, low complement 3, longer disease duration, hypertension, and the presence of mucocutaneous ulcers, as well as anticoagulants and steroid use, were among the important factors associated with SA in SLE patients. On the other hand, the administration of biologics and cyclosporine A and smoking were less important factors compared to the other selected features (Figure 2).

**Figure 3.**
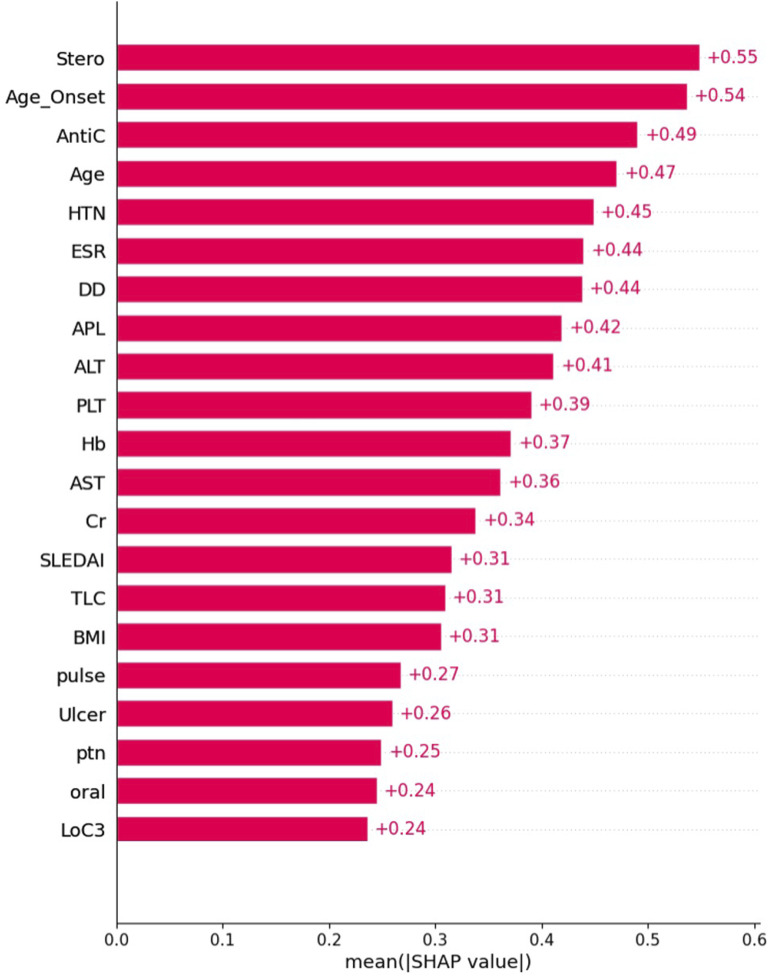
Variable contribution to the model by SHapley Additive exPlanation (SHAP) value; the individual contribution value of the top features.

Figure 3 represents the top most influential features to SA, including the steroid used, age at disease diagnosis, anticoagulant use, and hypertension status.

## Discussion

Predicting SA in patients with SLE can significantly aid in the provision of therapeutic and health consulting services that are effective in averting adverse outcomes associated with SA. However, identifying SA pattern is still complicated because of the multiplicity and complexity of affecting variables; using a machine ML, we achieved an AUC of 99%, indicating excellent discriminatory performance. Furthermore, using ML feature importance analysis, we ascertained the significant variables that exhibited an association with SA risk.

In the current study, the optimized XGBoost model identified patients at higher risk of SA in women with SLE better than LR algorithm (99% vs 78%, respectively). In a Chinese study,^11^ the authors retrospectively analyze 288 variables from 51 pregnant women with SLE to predict adverse pregnancy outcomes using six ML models. They found that ML models could compensate for the shortcomings of other statistical methods in situations where a small sample size issue arises in addition to the large number of variables that have been collected. Clinical practitioners may be more familiar with the predicted outcome by regression models, while sometimes ML may be helpful to operate the model nonlinear interactions among multiple clinical predictors more effectively than traditional statistical approaches.

Although, to the best of our knowledge, there are no other studies which used optimized XGBoost (HH-XGB) for predicting the pregnancy outcomes of SLE-affected women, researchers successfully applied other ML models for predicting pregnancy outcomes. A recent study exploited different ML models, including XGBoost, for predicting the risk of miscarriage among 656 patients with immune abnormalities including SLE and APS.^27^ Similar to our result, XGBoost demonstrated the best performance with an AUC of 0.921. Fazzari et al.^28^ evaluated whether ML approaches, in particular least absolute shrinkage and selection operator (LASSO), random forest (RF), support vector machines (SVM-RBF), gradient boosting (GB), and SuperLearner (SL), could improve adverse pregnancy outcome prediction in 385 subjects with SLE. SuperLearner performed the best (AUC = 0.78), but was statistically indistinguishable from LASSO, SVM, RBE and RF (AUC = 0.77 for all). Machine learning provides powerful tools for investigating SLE, a highly heterogeneous disease. Unlike conventional statistical methods,^29^ ML can identify complex and nonlinear interactions among multiple clinical variables.^30^

Pregnancies with SLE are associated with an increased risk of fetal problems, including SA.^31^ SA remains a significant concern in SLE pregnancies, with reported rates of 13.9–19.5% across studies, which is higher than the general population.^7,32^ In the present study, 13.9% of the included SLE patients had at least one SA. A retrospective cohort study conducted at a tertiary medical center in China included 338 pregnant women who had SLE and 1014 pregnant women who did not. The results showed that 3.3% of the women in the SLE group had spontaneous abortions.^33^ In another retrospective study conducted on 59 pregnant women with SLE (121 pregnancies), SA was observed in 38.8%.^34^ In a comprehensive analysis of 91 pregnancies in Egypt, Eman et al.^35^ documented a SA rate of 15%. There is variation in the frequency of SA in SLE among different studies and populations, which may be influenced by factors such as disease severity, treatment regimens, and genetic predispositions. Understanding these variations is crucial for improving prenatal care and outcomes for women with SLE. By identifying the specific factors that contribute to these differences, healthcare providers can tailor their approaches to better support affected individuals.

The second main finding of the present study is the feature selection used to identify informative variables. In most cases, the cause of a spontaneous abortion is unknown. The current work identified numerous risk factors for the pregnancy loss in women with SLE. On the one hand, some of these factors are non-lupus related and studies have shown a link between hypertension, positive antiphospholipid antibodies, steroid and anticoagulant use with a higher chance of pregnancy loss. Other identified risk factors were specific to SLE, including low complement 3 levels, longer disease duration, and the presence of mucocutaneous ulcers. A multi-factor regression analysis showed that the combined APS and SLEDAI were the main risk factors of fetal loss in SLE women.^14^ Moreover, Fazzari and colleagues confirmed, by using ML models, the role of antihypertensive medication use, low platelets, SLE disease activity, and lupus anticoagulant positivity as risk factors for adverse events during pregnancy.^28^

It was not surprising to find that positive antiphospholipid antibodies (aPLs) were a significant risk factor associated with SA in our SLE cohort. The main aPLs include anticardiolipin antibodies (aCLs), lupus anticoagulant (LA), and anti-β2-glycoprotein I antibodies (aβ2GPI).^36^ In general, APLs are identified as the main cause of recurrent SA.^37^ Additionally, the European League Against Rheumatism (EULAR) considers LA+ and triple positivity as high-risk aPL profiles for both thrombotic and adverse obstetric outcomes.^38^ The cross-immunity responses triggered by the antigen-antibody complexes can activate the complement system, generate inflammation reactions, and impede the growth and development of trophoblasts.^39^ Furthermore, these APLs have the ability to directly harm the placenta by disrupting the fusion, invasion, and proliferation of the trophoblastic layer. These factors are implicated in the occurrence of SA, placental disease, and fetal growth restriction.^40^

Hypocomplementemia has been estimated to account for roughly 20% of early pregnancy loss cases that are otherwise unexplained.^41^ In the current study, we identified low complement 3 as a risk factor associated with SA in SLE patients. The complement 3 was the main risk factors of increased SLE activity during pregnancy.^14^ In fact, there is conflicting data regarding the levels of complement components in women who experience early pregnancy loss. One study has found that greater levels of C3 and C4 can predict subsequent loss.^42^ However, other authors have reported that individuals with recurrent miscarriages may have hypocomplementemia.^43^ A novel predictive model for fetal loss was recently established by Wu et al. A retrospective analysis of 338 SLE pregnancies was conducted for this study. Consequently, C3 hypocomplementemia was identified as a significant independent risk factor associated with fetal loss.^8^ Also, Clowse et al. reported that low complement levels during the second trimester were associated with an increased risk of miscarriage and preterm births, irrespective of SLE activity.^44^ Animal models of repeated miscarriages showed that excessive complement activation is linked to poor angiogenesis and unfavorable pregnancy outcomes.^45^

Glucocorticoids are widely utilized to manage aggressive lupus activity during pregnancy.^15^ However, prolonged administration of glucocorticoids during pregnancy elevates the likelihood of adverse pregnancy outcomes. The study conducted by Reinisch et al. demonstrated a significant correlation between intrauterine growth restriction and the use of glucocorticoids, regardless of the presence of maternal disease activity.^46^ Previous studies have demonstrated that the use of steroids during pregnancy is a risk factor for preterm birth of and low birth weight.^47,48^ The results of the current study revealed that glucocorticoids administration is associated with SA. As detailed by Lateef and Petri^49^ prednisone can be used to manage disease activity in pregnancy, as most does not traverse the placenta, but its use should be minimized.

While systematic monitoring is recommended for all pregnant women with SLE to optimize outcomes, our findings suggest that those with identified risk factors associated with SA may require more frequent and targeted care. Thereby, awareness of the risk factors for the mothers can help to manage the condition more strictly by regular monitoring of the patient’s medical history and risk factors. Therefore, rheumatologists can decrease the effect of specific factors associated with the SA in SLE-affected women until delivery, and thus, it can maximize the chances of a successful delivery of live fetus.

In the current study, an optimized machine learning algorithm was applied to a large SLE cohort in order to achieve the best accurate model, and to identify the most relevant features by utilizing the decision-making logic of the tree. Several limitations should be considered in this study. While this study captured spontaneous abortion occurrences after SLE diagnosis, the precise interval between diagnosis and abortion was not recorded. In addition, we could not access data about anti Ro/SS-A and anti La/SS-B antibodies. Due to the cross-sectional design of the study, the temporal sequence between certain features and the outcome could not be strictly established. Specifically, variables such as therapeutic interventions (e.g., anticoagulants) might reflect a management response to a history of spontaneous abortion (reverse causality) rather than serving as risk factors. This strong association likely contributed to the high performance observed in our model. The ECR-SLE data collected obstetric history as a patient-level variable (presence or absence of ≥1 prior SA) and did not include pregnancy-specific data, thus, we were unable to distinguish women with multiple pregnancies or evaluate outcomes at the pregnancy level. Future studies should adopt pregnancy-level datasets or restrict analyses to first pregnancies to more accurately model SA risk. Finally, we replaced missing data using the regressive imputation method which might result in bias.

In conclusion, using information obtained during standard healthcare clinical practice, ML identified patterns of SA in women with SLE with high accuracy. Furthermore, the current work identified numerous risk factors such presence of mucocutaneous ulcers, longer disease duration, positive antiphospholipid antibodies, and low complement 3 for the pregnancy loss in women with SLE. Further longitudinal studies are necessary to evaluate the clinical utility of the proposed prediction model.

## Data Availability

The datasets used and/or analysed during the current study are available from the corresponding author on reasonable request.*
